# Educational technology on standard precautions for the knowledge, attitudes, and practices of Nursing students

**DOI:** 10.1590/0034-7167-2025-0023

**Published:** 2026-07-10

**Authors:** Suelen Gonçalves de Oliveira, Laurelize Pereira Rocha, Janaina Sena-Castanheira, Ana Paula Mousinho Tavares, Marlise Capa Verde Almeida de Mello

**Affiliations:** I Universidade Federal do Rio Grande. Rio Grande, Rio Grande do Sul, Brazil; II Universidade Federal de Pelotas. Pelotas, Rio Grande do Sul, Brazil

**Keywords:** Educational Technology, Education, Nursing, Health Knowledge, Attitudes, Practice, Universal Precautions, Students, Nursing., Tecnología Educacional, Educación en Enfermería, Conocimientos, Actitudes y Práctica en Salud, Precauciones Universales, Estudiantes de Enfermería.

## Abstract

**Objectives:**

to analyze the knowledge, attitudes, and practices of nursing students regarding standard precautions and the contribution of a digital guide to their training.

**Methods:**

this quantitative, cross-sectional, exploratory, and descriptive study was conducted in three stages with nursing students. In the first stage, of the Questionnaires for Knowledge and Compliance with Standard Precaution was applied; in the second, the digital guide was distributed; and in the third, an instrument developed to assess the contribution of this educational technology was used.

**Results:**

144 students participated in the first stage. Knowledge and adherence scores related to standard precautions were 83.3% and 89.9%, respectively. In the third stage, 56 students participated, with scores of 98.6%, 87.2%, and 94.6% for knowledge, attitudes, and practices, respectively.

**Conclusions:**

it is necessary to encourage students through diverse educational technologies to ensure safe patient care and reduce the risk of contamination and infection transmission.

## RESUMEN

**Objetivos:**

analizar el conocimiento, actitud y práctica de estudiantes de Enfermería sobre precauciones-estándar y la contribución de una guía digital para la formación.

**Métodos:**

estudio cuantitativo, transversal, exploratorio y descriptivo, realizado en tres etapas con estudiantes de Enfermería. En la primera etapa, se utilizó el *Questionnaires for Knowledge and Compliance with Standard Precaution*; en la segunda utilizamos la guía digital; y en la tercera, se utilizó un instrumento elaborado para analizar la contribución de la tecnología educacional.

**Resultados:**

en la primera etapa, participaron 144 estudiantes. Los puntajes de conocimiento y adhesión a las precauciones-estándar fueron 83,3% y 89,9%, respectivamente. En la tercera etapa, participaron 56 estudiantes, para verificar el conocimiento, la actitud y práctica, alcanzando 98,6%, 87,2% y 94,6%, respectivamente.

**Conclusiones:**

es necesario estimular a los estudiantes, con diferentes tecnologías educativas, para garantizar una asistencia segura a los pacientes y disminuir el riesgo de contaminación o de propagación de infecciones.

## INTRODUCTION

Healthcare-associated infections (HAIs) can affect patients, healthcare professionals, and students who are exposed to infectious agents during care delivery. To prevent the spread of microorganisms, specific measures must be implemented^(1)^, including a set of protocols known as standard precautions^(2)^. These precautions represent the minimum practices that must be followed when providing patient care^(1)^.

Standard precautions are addressed in undergraduate nursing programs; however, insufficient knowledge acquisition and inadequate implementation during training may result in poor compliance with these precautions, contributing to increased rates of HAIs^(3)^. Adequate knowledge grounded in scientific evidence, combined with positive attitudes and proper practices, is essential to ensure safe care^(4)^. Therefore, further research is needed to assess students’ knowledge, behaviors, and practices.

Studies assessing Knowledge, Attitudes, and Practices (KAP) provide specific information that helps guide targeted interventions aimed at improving problem-solving capacity and achieving effective outcomes, whenever possible^(5)^. Therefore, these studies are often used by researchers in the health field^(6)^. In the context of academic training, identifying knowledge gaps is essential to promote the necessary changes^(7)^.

It is well established that the use of educational technologies is an important strategy to support professional training. When applied appropriately, these tools can foster meaningful transformations, enhance knowledge and skills, and promote high-quality practice^(8,9)^. Various technologies can be recommended for this purpose^(10)^. Digital educational technologies, for instance, make learning more engaging and expand access to knowledge^(11)^. Among these, digital guides stand out as low-cost, easily accessible resources available anytime and anywhere^(10)^. Therefore, this study was developed considering the importance of compliance with standard precautions across the domains of knowledge, attitudes, and practices.

## OBJECTIVES

To analyze nursing students’ knowledge, attitudes, and practices regarding standard precautions, as well as the contribution of a digital guide to their training.

## METHODS

### Ethical aspects

This study is part of the macroproject titled “Knowledge, attitudes, and practices of nursing students regarding standard precautions mediated by educational technology”. The ethical principles governing research involving human participants were followed, in accordance with Resolution No. 466 of 2012. Additionally, the study was approved by the Institutional Review Board in August 2023.

### Study design, period and setting

This quantitative, cross-sectional, exploratory, and descriptive study followed the Strengthening the Reporting of Observational Studies in Epidemiology (STROBE) guidelines. The study was conducted in three phases, between July and October 2024, in an undergraduate nursing program at a federal university in southern Brazil. The program, established in 1975, currently admits students through the Unified Selection System (SISU), based on scores from the National High School Exam (ENEM), and consists of 10 semesters of full-time training.

### Population, sample calculation, and selection criteria

This study included students from the 2^nd^ to 9^th^ semesters of the undergraduate nursing program. The study began with students in the second semester because it marks their initial contact with the nursing profession. Students in the 10^th^ semester were not included, as they were only 10 days away from graduation. Moreover, because the study included a later phase, those students would not have been available for the second data collection. The difference in the academic calendar between the 10^th^ semester and the other semesters was due to environmental factors resulting from extreme weather events in the region where the study was conducted.

The sample size calculation was performed using Statcalc (Epi Info™, version 7.2.1.0). There were 218 students enrolled between the 2^nd^ and 9^th^ semesters. Considering a 95% confidence interval and a 5% margin of error, the minimum sample size was 138 students. Convenience sampling was used, with the following inclusion criterion: being formally enrolled from the 2^nd^ to 9^th^ semester. Exclusion criteria included students with suspended enrollment, those on sick leave, or those transferring from another institution. In addition, three students who participated in the pilot study-aimed at minimizing errors in the second-stage instrument and estimating questionnaire response time-were excluded.

The researchers used three approaches to invite the students to participate: in-person invitations to those present in class and invitations sent via WhatsApp and email. The survey was available in both paper and electronic formats. Printed invitations containing a QR code for online access to the survey were also distributed. A participant was considered lost to follow-up when, after three attempts through each of the three communication channels (in person, WhatsApp, and email) no response was received.

### First stage

Initially, the Questionnaires for Knowledge and Compliance with Standard Precautions-originally developed and validated in China^(12)^ and later adapted and validated for Brazilian Portuguese-was administered. The instrument consists of three questionnaires: one for sociodemographic analysis and two others to assess knowledge and adherence to standard precautions^(13-15)^.

The questionnaire assessing knowledge of standard precautions (QCPP) consists of 20 items rated on a Likert scale: “True” (1 point), “False” (0 points), and “I don’t know” (0 points). The total score ranges from 0 to 20 points^(13-14)^. The questionnaire assessing adherence to standard precautions (QAPP) also includes 20 items, rated on a five-point Likert scale: “Always” (4 points), “Usually” (3 points), “Sometimes” (2 points), “Seldom” (1 point), and “Never” (0 points). The total score ranges from 0 to 80 points^(13,15)^. Scores on both the QCPP and QAPP are directly related to knowledge and adherence to standard precautions: the higher the score, the greater the knowledge and adherence^(13)^. Scores ≥75% on each instrument were considered indicative of “good knowledge” and “good adherence”.

A total of 144 students participated in this stage. Those who agreed to participate signed two copies of an informed consent form-one retained by the researcher and the other by the student. For the online format, students signed the form digitally and received a copy, also signed by the researcher, via email. After consent, the sociodemographic questionnaire adapted by the researchers was administered to characterize the target population, followed by the QCPP and QAPP. This stage lasted 24 days. In the pilot study, the questionnaire response time ranged from 8 to 12 minutes.

### Second stage

After completing the first stage, the researchers sent students a digital guide titled *“Precauções-padrão para Serviço de Saúde*” [Standard Precautions for Healthcare Services] via WhatsApp and email. The guide addressed topics such as hand hygiene, personal protective equipment (PPE), waste segregation and disposal, accidents involving biological material, and surface cleaning and disinfection. It included concise information to guide best practices; short videos or step-by-step instructions illustrating recommended procedures; and reflection questions designed to encourage critical thinking on the topic. The content was developed based on the theoretical gaps identified in the results of the study’s first stage. It was also informed by a scoping review-whose protocol was registered on the Open Science Framework platform (DOI 10.17605/OSF.IO/Z6KW)-that mapped the knowledge, attitudes, and practices of nursing students and professionals regarding standard precautions in a hospital setting. The writing was supported by scientific studies and official Brazilian and international references, including those from the World Health Organization (WHO), the Brazil’s *Agência Nacional de Vigilância Sanitária* (Anvisa) [National Health Surveillance Agency], and the Centers for Disease Control and Prevention (CDC), among others.

The digital guide was developed by the researchers and validated by 11 experts from five different universities: the Federal University of Rio Grande, the Federal University of Pelotas, the Federal University of Health Sciences of Porto Alegre, the Federal University of Santa Catarina, and the University of São Paulo. These experts specialized in patient safety, educational technology, healthcare-associated infections, and the KAP methodology.

When the digital guide was distributed, students were instructed to read it and compare their prior knowledge with the information presented, identifying practices that should be adjusted after reading. The researchers informed them that they would be contacted again in 14 days and invited to participate in the study’s final stage.

### Third stage

Data collection for this stage lasted 40 days and included 56 students. In the pilot study, the average questionnaire completion time was nine minutes. The instrument, developed by the authors, was administered in two sections: the first contained sociodemographic questions, and the second included questions about the digital guide. Of the 28 questions about the guide, 21 were objective, with “Yes”, “No”, and “I don’t know” answer options, referring to students’ observations about the content regarding knowledge, attitudes, and practices. Seven were multiple-choice questions addressing the topics covered in each chapter of the guide.

One of the questions specifically addressed hand hygiene and sought to identify which topics had been most meaningful or had provided new information for the students. The answer options were: “Hand hygiene moments”, “Hand hygiene techniques (with alcohol-based hand rub or soap and water)”, “Hand care”, and “I don’t know”.

The remaining six questions addressed PPE and sought to identify which information had been most meaningful or had provided new insights. The answer options for these questions were: “Indication for use”, “How to put on and/or remove”, “Disposal location”, “How to clean and disinfect”, and “I don’t know”. Finally, three optional open-ended questions were included to allow students to comment on the digital guide they received or the study itself.

### Data analysis

The data were tabulated in Microsoft Excel^®^ and exported to the Statistical Package for the Social Sciences (SPSS^®^), version 22. In Excel, the QCPP and QAPP scores were analyzed. In SPSS, the data were analyzed using descriptive statistics, including absolute and relative frequencies and standard deviation.

## RESULTS

In the first stage of the study, 144 students participated: 12 (8.3%) were in the 2^nd^ semester; 23 (16%) in the 3^rd^ semester; 18 (12.5%) in the 4^th^ semester; 18 (12.5%) in the 5^th^ semester; 14 (9.7%) in the 6^th^ semester; 24 (16.7%) in the 7^th^ semester; 18 (12.5%) in the 8^th^ semester; and 17 (11.8%) in the 9^th^ semester.

Regarding the assessment of students’ knowledge, the mean score was 16.67, corresponding to 83.3% correct answers, which is considered a good level of knowledge. The results showed a predominance of correct answers to questions related to hand hygiene and glove use. When analyzing the remaining questions, only 28.5% of participants were aware of the need to implement airborne precautions, in addition to standard precautions, when providing nursing care to patients with chickenpox or active tuberculosis. The other results related to students’ knowledge of standard precautions are presented in Table 1.

**Table 1:** Nursing students’ knowledge of standard precautions (N = 144), Rio Grande, Rio Grande do Sul, Brazil, 2024

Item	True n (%)	False n (%)	I do not know n (%)	Total N (%)
Do you know what standard precautions are?	127 (88.2)	0 (0)	17 (11.8)	144 (100)
Standard precautions should only be applied to patients diagnosed with an infection or those in the incubation period of a given infection.	17 (11.8)	119 (82.6)	8 (5.6)	144 (100)
Adherence to standard precautions aims primarily to protect healthcare personnel.	110 (76.4)	28 (19.4)	6 (4.2)	144 (100)
If you come into contact with blood or any other potentially contaminated material, wash your hands immediately.	140 (97.2)	2 (1.4)	2 (1.4)	144 (100)
Hand hygiene should be performed when providing care to different patients.	135 (93.8)	8 (5.6)	1 (0.7)	144 (100)
Since wearing gloves prevents hand contamination, there is no need to clean your hands after removing them.	4 (2.8)	140 (97.2)	0 (0)	144 (100)
Contact with objects, materials, equipment, clothing, or individuals wearing contaminated PPE must be avoided.	141 (97.9)	2 (1.4)	1 (0.7)	144 (100)
PPE should not be shared.	138 (95.8)	4 (2.8)	2 (1.4)	144 (100)
When performing oral care or other procedures that may involve contact with a patient’s mucous membranes, wearing gloves is not mandatory.	2 (1.4)	142 (98.6)	0 (0)	144 (100)
During blood collection or venipuncture procedures, wearing gloves is necessary.	143 (99.3)	1 (0.7)	0 (0)	144 (100)
When there is a possibility of hand contact with a patient’s secretions or excretions, wearing gloves is necessary.	144 (100)	0 (0)	0 (0)	144 (100)
Gloves should be changed when caring for different patients.	144 (100)	0 (0)	0 (0)	144 (100)
In procedures where there is a possibility of splashing blood, body fluid, secretions, or excretions, a mask or face shield must be used.	142 (98.6)	1 (0.7)	1 (0.7)	144 (100)
In procedures where there is a possibility of splashing blood, body fluid, secretions, or excretions, protective goggles or a face shield must be used.	140 (97.2)	2 (1.4)	2 (1.4)	144 (100)
In procedures where there is a possibility of splashing blood, body fluid, secretions, or excretions, a waterproof protective gown must be used.	140 (97.2)	1 (0.7)	3 (2.1)	144 (100)
In situations where there is a possibility of splashing blood, body fluid, secretions, or excretions, disposable caps and shoe covers must be used.	114 (79.2)	14 (9.7)	16 (11.1)	144 (100)
It is forbidden to bend, twist, or actively recap needles. When necessary, perform passive recapping with one hand only. Disposal containers must be placed close to the handling area.	109 (75.7)	19 (13.2)	16 (11.1)	144 (100)
When providing nursing care to patients with hepatitis C or syphilis, only standard precautions are required.	82 (56.9)	34 (23.6)	28 (19.4)	144 (100)
When providing nursing care to patients with active tuberculosis or chickenpox, standard precautions must be used in addition to droplet precautions.	92 (63.9)	41 (28.5)	11 (7.6)	144 (100)
When providing nursing care to patients with intestinal or skin infections, standard precautions must be used in addition to contact precautions.	124 (86.1)	9 (6.3)	11 (7.6)	144 (100)

Regarding students’ adherence to standard precautions, the mean score was 71.5 points (89.9%), which is considered a good adherence rate. This score, as well as the knowledge score, was again supported by glove use practices, which achieved over 90% correct answers in most questions on this topic. The use of protective eyewear, a cap, and a gown when there was a possibility of exposure to blood splashes, body fluids, secretions, or excretions showed adherence rates of 48.6%, 38.9%, and 53.3%, respectively. Other results regarding adherence to standard precautions are presented in Table 2.

**Table 2:** Nursing students’ adherence to standard precautions (N = 144), Rio Grande/Rio Grande do Sul, Brazil, 2024

Item	Always n (%)	Usually n (%)	Sometimes n (%)	Seldom n (%)	Never n (%)	Total N (%)
I perform hand hygiene between providing care to different patients.	115 (79.9)	25 (17.4)	2 (1.4)	1 (0.7)	1 (0.7)	144 (100)
I perform hand hygiene after removing gloves.	111 (77.1)	26 (18.1)	6 (4.2)	0 (0)	1 (0.7)	144 (100)
I wash my hands immediately after contact with potentially contaminated biological material.	138 (95.8)	4 (2.8)	1 (0.7)	0 (0%)	1 (0.7)	144 (100)
Report **the frequency of glove use** in procedures where there is a possibility of contact with potentially contaminated biological material, listed below.						
Blood collection^*^	139 (96.5)	3 (2.1)	0 (0)	0 (0)	1 (0.7)	143 (99.3)
Procedures involving possible contact with urine or feces^*^	138 (95.8)	4 (2.8)	0 (0)	0 (0)	1 (0.7)	143 (99.3)
Procedures involving possible contact with the patient’s non-intact skin^*^	131 (91.0)	9 (6.3)	2 (1.4)	0 (0)	1 (0.7)	143 (99.3)
Procedures involving possible contact with the patient’s mucous membranes^*^	135 (93.8)	4 (2.8)	3 (2.1)	0 (0)	1 (0.7)	143 (99.3)
Procedures involving possible contact with the patient’s airway secretions ^*^	135 (93.8)	5 (3.5)	2 (1.4)	0 (0)	1 (0.7)	143 (99.3)
Intramuscular or subcutaneous injection ^*^	107 (74.3)	23 (16.0)	9 (6.3)	2 (1.4)	2 (1.4)	143 (99.3)
Dressing procedures ^**^	137 (95.1)	4 (2.8)	0 (0)	0 (0)	1 (0.7)	142 (98.6)
Cleaning to remove blood ^**^	135 (93.8)	5 (3.5)	0 (0)	1 (0.7)	1 (0.7)	142 (98.6)
Venipuncture^****^	132 (91.7)	7 (4.9)	0 (0)	0 (0)	1 (0.7)	140 (97.2)
Contact with blood samples ^**^	130 (90.3)	10 (6.9)	1 (0.7)	0 (0)	1 (0.7)	142 (98.6)
I use a protective mask when there is a possibility of exposure to splashes of blood, body fluids, secretions, or excretions.	112 (77.8)	18 (12.5)	9 (6.3)	2 (1.4)	3 (2.1)	144 (100)
I use protective goggles when there is a possibility of exposure to splashes of blood, body fluids, secretions, or excretions.	70 (48.6)	25 (17.4)	25 (17.4)	19 (13.2)	5 (3.5)	144 (100)
I use a protective gown when there is a possibility of exposure to splashes of blood, body fluids, secretions, or excretions.	77 (53.5)	38 (26.4)	19 (13.2)	9 (6.3)	1 (0.7)	144 (100)
I use disposable caps and shoe covers when there is a possibility of exposure to splashes of blood, body fluids, secretions, or excretions.	56 (38.9)	31 (21.5)	34 (23.6)	15 (10.4)	8 (5.6)	144 (100)
I do not actively recap used needles and, when necessary, perform passive recapping using only one hand.	95 (66.0)	23 (16.0)	6 (4.2)	6 (4.2)	14 (9.7)	144 (100)
I dispose of needles, blades, and other sharps in designated disposal containers.	138 (95.8)	3 (2.1)	1 (0.7)	0 (0)	2 (1.4)	144 (100)
After occupational accidents involving contaminated sharps, I immediately squeeze the affected area, then wash it, disinfect it, and apply a dressing. ^***^	64 (44.4)	20 (13.9)	4 (2.8)	10 (6.9)	43 (29.9)	141 (97.9)

* One participant did not answer this item;

** two participants did not answer this item;

*** three did not answer this item;

**** four did not answer this item.

The scores of 17/20 for knowledge and 72/80 for adherence were positive; however, a detailed analysis of the questionnaire responses revealed some gaps. Although 88.2% of students reported knowing what standard precautions are, only 56.9% agreed that these are sufficient for providing care to patients with hepatitis C or syphilis. Furthermore, 64.3% of students agreed that airborne precautions, in combination with standard precautions, should be adopted when caring for patients with chickenpox or active tuberculosis; and 66% stated that they always dispose of used needles without recapping them.

At the end of the first stage, the digital guide was distributed to the students. Fourteen days later, the third stage began, involving 56 participants: one from the 2^nd^ and one from the 3^rd^ semester; two from the 4^th^ and two from the 5^th^ semester; seven from the 6^th^ semester; 18 from the 7^th^ semester; 15 from the 8^th^ semester; and 10 students from the 9^th^ semester. Of these, 92.9% were between 18 and 30 years old.

After receiving the digital guide, 96.4% of students reported that it improved their knowledge, 87.5% indicated a change in attitude based on it, and 92.9% stated that it improved their practice. Among the topics covered, the most significant for students was the chapter on hand hygiene, with 100% agreement that this content helped improve their knowledge. “Hand hygiene moments” and “Hand hygiene techniques” were the topics that contributed most to knowledge improvement, with 73.2% and 57.1% agreement among students, respectively. The other topics also achieved high agreement regarding knowledge improvement. Information on glove use, protective eyewear, caps, accidents with biological material, and surface cleaning and disinfection improved the knowledge of 98.2% of students. Regarding changes in practice after reading the guide, hand hygiene and surface cleaning and disinfection were the actions most frequently reported as improved, with 87.5% in both cases. Next, 85.7% reported changes in waste segregation practices. In contrast, the lowest number of positive responses referred to the use of protective eyewear or face shields, with 62.5% of students reporting increased frequency of use.

## DISCUSSION

Standard precautions must be an integral part of nursing education. They should be incorporated into the curriculum and addressed systematically and across disciplines^(16,17)^. Strengthening nursing students’ knowledge is a prerequisite for safe practice^(16)^, both in synchronous learning and through diverse educational resources and tools^(18)^. Theoretical knowledge enables students to broaden their perception and make sound clinical decisions^(19)^. Self-report surveys have been widely used to assess knowledge of and adherence to standard precautions^(18)^. Although these studies have shown generally satisfactory results, it is essential to explore participants’ knowledge in depth to ensure that positive findings do not conceal weaknesses in specific areas^(20)^.

This study reflected a knowledge-focused approach by identifying discrepancies between high knowledge scores and low adherence in supplementary questions. For instance, some students reported knowing when to apply standard precautions but were unable to identify the necessary actions in specific situations, such as providing care to patients with chickenpox or active tuberculosis. This finding indicates that more than 30% of students lacked knowledge about infection transmission routes and the appropriate use of PPE for each type of exposure.

Chickenpox and active tuberculosis are airborne diseases and therefore require the use of N95 or PFF2 respirators-the only devices capable of protecting the respiratory tract from microparticle exposure^(21,22)^. A study conducted in India with nursing and medical students demonstrated a similar gap in knowledge, showing that only 14% of nursing students selected the N95 respirator as the appropriate equipment for protection against airborne diseases and for use in aerosol-generating procedures^(3)^. For PPE to be appropriately selected according to its intended use and care provided, a proper assessment of exposure risks is essential. If such an assessment is inaccurate, compliance with protective measures is compromised, potentially leading to occupational exposure^(3)^. PPE is a fundamental resource in infection prevention^(20)^; however, despite its essential role, this study revealed low adherence to most types of equipment, except for gloves. The percentages of students who reported always wearing a cap, protective eyewear, and a gown were 51.8%, 55.4%, and 68.3%, respectively. Nonetheless, 100% of students reported being aware of the recommendation to wear protective eyewear, 98.2% were aware of the need for a gown, and 85.7% were aware of the recommendation to use a cap, indicating that this situation does not stem from a lack of knowledge.

A study conducted in Saudi Arabia and another in Brazil reported similar findings regarding the highest and lowest rates of PPE adherence. In both studies, the highest adherence was to gloves, followed by masks, while the lowest adherence was to gowns and protective eyewear or face shields^(23,24)^. Gloves also showed the highest self-reported adherence in the present study, ranging from 87.5% to 100% for most recommended occasions of use. In this case, adherence and knowledge were consistent, as 100% of the students were aware of the need to use this equipment when hands might come into contact with patients’ secretions or excretions, and they were also aware of the need to change gloves when providing care to different patients.

Glove use appears to be strongly embedded in care recommendations, while other equipment tends to be perceived as necessary only for activities with a higher risk of exposure^(25)^. However, exposure to potentially infectious agents can occur at similar rates across different routes of contact. Therefore, when facing fluid splashes, the risk of contamination of the eyes, nose, or mouth should not be overlooked, since, regardless of the route of exposure, accidents involving potentially infectious materials can have serious consequences for healthcare workers^(24)^. Accidents involving biological material through mucous membrane exposure are a real concern, as demonstrated by a study conducted in Minas Gerais in which 38% of the 111 reported accidents involved mucous membrane exposure-events that could have been prevented through the use of appropriate equipment^(26)^.

However, in clinical settings, it is recognized that various professionals influence students. Therefore, low adherence to certain equipment may result from observing that other professionals do not use these resources. Students may be misled into believing that if qualified professionals do not use a particular item in their practice, it is because its use is unnecessary^(20)^. Thus, the presence of negative role models is understood to significantly hinder students’ adherence to standard precautions^(25)^. This problem requires early interventions to reinforce recommendations for standard precautions and to promote proper compliance.

To reduce this influence, educators play a crucial role in guiding and directing students toward best practices. However, for this role to be effective, they must serve as models of compliance with standard precautions^(25)^. Educators can also adopt strategies such as frequently reminding students during clinical activities about the measures they must follow to prevent infections^(16)^ or periodically encouraging reflection on the importance of adhering to standard precautions^(25)^. These behaviors are effective in strengthening students’ adherence to standard precautions^(27)^.

Reinforcing the importance of these measures can occur both through monitoring actions combined with feedback and through theoretical approaches. When organizational leadership that is visible to students is paired with strategies to promote high levels of adherence, the application of theoretical knowledge is strengthened^(27)^. Among the strategies that have shown positive results in this regard is the integration of technology into the teaching and learning process, given its potential to enhance or even accelerate student development. Much of this benefit is influenced by the current student profile. Therefore, identifying which generation predominates in higher education is essential when designing educational models^(28)^. Currently, the most prevalent generations among higher education students are Generation Y and Generation Z^(29)^, as indicated by the study results showing that 94.6% of participants were between 18 and 40 years old.

Despite their particularities, both generations use technology. Generation Z students, as the first generation born into an era of widespread technology use^(28,29)^, demonstrate significantly greater development in this area. In higher education, these students prefer to learn through electronic and audiovisual materials. Resources such as WhatsApp are widely adopted by this group because they facilitate the sharing of academic information among peers. The main appeal for them is the constant availability of digital resources^(28)^. Therefore, recognizing this generation’s preferences and the importance of technology in education, the study developed a digital guide, which was considered an appropriate strategy to enhance knowledge and encourage greater adherence to standard precautions.

Using technology in the educational process has shown advances-albeit modest-in learning outcomes related to the intervention^(28)^. This was also observed in this study, with positive results following the distribution of the digital guide. Its objective and interactive content contributed to students’ theoretical and practical learning, enhancing knowledge, encouraging positive attitudes, and guiding practice. Sustaining the skills necessary for infection prevention and control requires effort, time^(17)^, and awareness of exposure risks, which is a key factor influencing adherence to standard precautions^(30)^. Possibly for this reason, agreement that the digital guide encouraged positive attitudes was the lowest among the three assessments. Even so, it reached 87.2%, slightly below the percentages attributed to improvements in knowledge and practice.

The time between acquiring knowledge from the guide and applying that knowledge in practice may have influenced students’ understanding of risks and their perception of being at risk. Therefore, further studies are needed to compare self-reported behavior with observed practice. This strategy should continue to be applied and expanded among students.

### Study limitations

This study was conducted in the state of Rio Grande do Sul, Brazil, during an atypical academic calendar affected by extreme weather events. These circumstances led to numerous interruptions in academic activities and reduced the time available for research, significantly affecting the third phase. Another limitation was the low participation of students in this phase-especially those in early semesters-which prevented comparisons between semesters. This may be explained by factors such as limited knowledge about research development, insufficient exposure to related content in theoretical courses, and limited practical experience in healthcare settings. These limitations indicate that pedagogical adjustments are needed within the program and that further studies should be conducted to allow for broader comparisons.

### Contributions for the field

This study provided a digital guide on standard precautions, aiming to enhance knowledge and promote greater adherence to these measures. Furthermore, it identified several strategies to effectively implement the necessary actions to minimize the occupational exposure experienced by students.

## CONCLUSIONS

This study identified knowledge gaps, strategies to foster positive attitudes, practices that were consistent with recommendations, and those requiring intervention. The digital guide made positive contributions to students’ learning. Therefore, we recommend the continued use of diverse educational technologies throughout the training process to address potential doubts and promote nursing students’ adherence to standard precautions. Additionally, encouraging critical thinking by highlighting the risks and consequences of non-compliance can help raise awareness of the importance of each standard precaution guideline.

## Data Availability

The research data are available within the article.
