# Supplementary material for: Educational technology on standard precautions for the knowledge, attitudes, and practices of Nursing students
Source: Rev Bras Enferm. 2026 Jul 10;79:e20250023. doi: 10.1590/0034-7167-2025-0023 (PMC13363301; doi:10.1590/0034-7167-2025-0023)
Supplement: Translation

# Translation: Tecnologia educacional sobre precauções-padrão para o conhecimento, atitude e práticas de estudantes de Enfermagem

**DOI:** 10.1590/0034-7167-2025-0023pt

**Authors:** Suelen Gonçalves de Oliveira, Laurelize Pereira Rocha, Janaina Sena-Castanheira, Ana Paula Mousinho Tavares, Marlise Capa Verde Almeida de Mello

**Affiliations:** I Universidade Federal do Rio Grande. Rio Grande, Rio Grande do Sul, Brasil; II Universidade Federal de Pelotas. Pelotas, Rio Grande do Sul, Brasil

**Keywords:** Tecnologia Educacional, Educação em Enfermagem, Conhecimento, Atitude e Práticas em Saúde, Precauções Universais, Estudantes de Enfermagem.

## Abstract

**Objetivos:**

analisar o conhecimento, atitude e prática de estudantes de Enfermagem sobre as precauções-padrão e a contribuição de um guia digital para a formação.

**Métodos:**

estudo quantitativo, transversal, exploratório e descritivo, realizado em três etapas com estudantes de Enfermagem. Na primeira etapa, utilizou-se o *Questionnaires for Knowledge and Compliance with Standard Precaution*; na segunda, disponibilizou-se o guia digital; e na terceira, utilizou-se um instrumento elaborado para analisar a contribuição da tecnologia educacional.

**Resultados:**

na primeira etapa, participaram 144 estudantes. Os escores de conhecimento e adesão às precauções padrão foram 83,3% e 89,9%, respectivamente. Na terceira etapa, participaram 56 estudantes, verificando-se conhecimento, atitude e prática, alcançando 98,6%, 87,2% e 94,6%, respectivamente.

**Conclusões:**

é preciso estimular os estudantes com diferentes tecnologias educativas para garantir uma assistência segura aos pacientes, diminuindo o risco de contaminação ou propagação de infecções.

## INTRODUÇÃO

As infecções relacionadas à assistência à saúde (IRAS) podem acometer pacientes, profissionais e estudantes da área da saúde devido à exposição a agentes infecciosos durante a assistência. Para que os microrganismos não se propaguem, é necessário assumir determinadas ações^(1)^, que constituem um conjunto de protocolos denominados de precauções-padrão^(2)^. Elas correspondem às práticas mínimas que devem ser adotadas ao prestar assistência ao paciente^(1)^.

O tema das precauções-padrão é abordado no curso de graduação em Enfermagem. No entanto, quando há fragilidade na aquisição do conhecimento e execução inadequada das medidas durante a formação, isso pode levar à má conformidade das precauções-padrão, desencadeando o aumento das taxas de IRAS^(3)^. O adequado conhecimento embasado em fontes científicas confiáveis somado a atitudes positivas e boas práticas são essenciais para garantir a segurança da assistência^(4)^. Por essa razão, é importante desenvolver estudos que avaliem o que os estudantes conhecem, assumem como comportamento e praticam.

Pesquisas que avaliam o Conhecimento, Atitude e Prática (CAP) tendem a fornecer informações pontuais necessárias para direcionar, de forma assertiva, intervenções buscando a resolutividade, quando possíveis^(5)^. Por isso, essas pesquisas vêm sendo frequentemente utilizadas por pesquisadores da área da saúde^(6)^. Considerando a formação acadêmica, é ideal reconhecer lacunas no conhecimento para promover as mudanças necessárias^(7)^.

Sabe-se que, atualmente, a oferta de tecnologias educacionais é uma das formas de colaborar com a formação profissional. Quando utilizadas adequadamente, elas podem consolidar transformações promissoras, qualificar o conhecimento e as habilidades, além de favorecer uma prática qualificada^(8,9)^. Diversas tecnologias podem ser indicadas para esse fim^(10)^. As tecnologias educacionais digitais, por exemplo, tornam a aprendizagem mais atrativa e expandem o acesso ao conhecimento^(11)^. Entre elas, destaca-se o guia digital, um recurso de baixo custo e de fácil acesso a qualquer momento e lugar^(10)^. Dessa forma, este estudo foi desenvolvido considerando a importância da conformidade com as precauções-padrão nos âmbitos do conhecimento, da atitude e da prática.

## OBJETIVOS

Analisar o conhecimento, atitude e prática de estudantes de Enfermagem acerca das precauções-padrão e a contribuição de um guia digital para a formação.

## MÉTODOS

### Aspectos éticos

Este estudo faz parte do macroprojeto intitulado “Conhecimentos, atitudes e práticas de acadêmicos de Enfermagem acerca das precauções-padrão mediados por uma tecnologia educacional”. Foram respeitados os preceitos éticos da pesquisa com seres humanos, alinhando-se à Resolução n.º 466, de 2012, tendo sido aprovado pelo Comitê de Ética em Pesquisa em agosto de 2023.

### Desenho, período e local de estudo

Estudo quantitativo, transversal, exploratório e descritivo, que seguiu as recomendações das diretrizes *Strengthening the Reporting of Observational Studies in Epidemiology* (STROBE). Foi desenvolvido em três etapas, entre julho e outubro de 2024, em um curso de graduação em Enfermagem de uma universidade federal no Sul do país. Esse foi criado em 1975; e atualmente o ingresso nele é feito por meio do Sistema de Seleção Unificado (SISU), com base nas notas do Exame Nacional do Ensino Médio (ENEM). Conta com 10 semestres de formação em período integral.

### População, cálculo amostral e critérios de seleção

Este estudo foi realizado com estudantes do 2º ao 9º semestre do curso de graduação em Enfermagem. A escolha de iniciar a pesquisa com o 2º semestre deve-se ao fato de ser o primeiro momento de aproximação do estudante com o ser e fazer da profissão. Os estudantes do 10º semestre não foram incluídos porque estavam a apenas 10 dias de sua formatura e, como a pesquisa previa etapa posterior, estes não seriam incluídos para a segunda amostra. A diferença de calendário acadêmico entre o 10º semestre e os demais se deu por questões ambientais, em decorrência dos eventos climáticos extremos ocorridos na área onde a pesquisa se desenvolveu.

O cálculo amostral foi realizado por meio do *Statcalc* (Epi Info^TM^, versão 7.2.1.0). O curso contava com uma população de 218 estudantes matriculados entre o 2º e o 9º semestres; assim, considerando os parâmetros de 95% de confiança e 5% de chance de erro, a amostra mínima foi de 138 estudantes. Utilizou-se a amostragem por conveniência, seguindo os critérios de inclusão: ser aluno do 2º ao 9º semestre, devidamente matriculado no curso. Foram critérios de exclusão: estudantes com sistema de trancamento, em licença de saúde ou ainda em período de transferência entre instituições. Além disso, foram excluídos três estudantes que participaram do estudo-piloto, desenvolvido com os propósitos de minimizar erros no instrumento da segunda etapa e calcular o tempo de resposta dos questionários.

Para convidar os estudantes a participar, os pesquisadores utilizaram três meios: presença em sala de aula, WhatsApp e e-mail. A pesquisa foi disponibilizada em formato físico e eletrônico. Também foram distribuídos convites impressos contendo QR CODE para acesso on-line à pesquisa. Considerou-se perda quando, após três tentativas de contato em cada uma das três vias de comunicação (presencial, WhatsApp e e-mail), o estudante não respondeu.

### Primeira etapa da pesquisa

No primeiro momento, foi aplicado o instrumento *Questionnaires for Knowledge and Compliance with Standard Precaution*, originalmente elaborado e validado na China^(12)^ e posteriormente adaptado e validado para a língua portuguesa do Brasil. O instrumento contém três questionários: um para análise sociodemográfica e outros dois para avaliação do conhecimento e da adesão às precauções-padrão^(13-15)^.

O questionário que avalia o conhecimento sobre as precauções-padrão (QCPP) conta com 20 questões de respostas em escala Likert do tipo “Verdadeiro” (1 ponto), “Falso” (0 ponto) e “Não sei” (0 ponto). O escore máximo é de 20 pontos, enquanto o mínimo é 0^(13,14)^. O questionário de adesão às precauções-padrão (QAPP) contém também 20 questões, mas com respostas em escala Likert de cinco pontos: “Sempre” (4 pontos), “Frequentemente” (3 pontos), “Às vezes” (2 pontos), “Raramente” (1 ponto) e “Nunca” (0 ponto). O escore varia de 0 a 80 pontos^(13,15)^. A pontuação, tanto no QCPP quanto no QAPP, está diretamente relacionada ao conhecimento e às adesões sobre as precauções-padrão. Quanto maior o escore, maiores são o conhecimento e a adesão da população^(13)^. Consideraram-se como “bom conhecimento” e “boa adesão” escores ≥75%, em cada instrumento.

Participaram desta etapa 144 estudantes. Aquele com interesse em participar assinou o Termo de Consentimento Livre e Esclarecido (TCLE) em duas vias impressas, ficando uma com a pesquisadora e a outra com o estudante. Quando no formato on-line, o estudante o assinava digitalmente e recebia via e-mail uma cópia do TCLE também assinado pela pesquisadora responsável. Após essa etapa, havia o questionário sociodemográfico adaptado pelas pesquisadoras, para a caracterização do público-alvo; na sequência estavam, respectivamente, os instrumentos QCPP e QAPP. Essa etapa durou 24 dias. No estudo-piloto, o tempo de resposta ao questionário variou de 8 a 12 minutos.

### Segunda etapa da pesquisa

Depois da finalização da primeira etapa, as pesquisadoras encaminharam aos estudantes, via WhatsApp e e-mail, um guia digital intitulado “Precauções-padrão para Serviço de Saúde”. Ele aborda conteúdos de higiene de mãos, equipamentos de proteção individual (EPIs), segregação e descarte de resíduos, acidente com material biológico e limpeza e desinfecção de superfícies. O guia traz informações objetivas orientando boas práticas; vídeos curtos ou um passo a passo ilustrando práticas recomendadas; e questionamentos para gerar reflexões sobre a temática. O conteúdo foi elaborado com base nas fragilidades teóricas identificadas nos resultados da primeira etapa de pesquisa. Fundamentou-se também em uma revisão de escopo - cujo protocolo foi registrado na plataforma *Open Science Framework* (DOI 10.17605/OSF.IO/Z6KW) - que mapeou o conhecimento, as atitudes e as práticas de acadêmicos e profissionais de enfermagem acerca das precauções-padrão no contexto hospitalar. Para a escrita, foram utilizados estudos científicos e referências oficiais nacionais e internacionais, como a Organização Mundial de Saúde (OMS), Agência Nacional de Vigilância Sanitária (Anvisa), *Centers For Disease Control and Prevention* (CDC), entre outras.

O guia digital foi elaborado pelas pesquisadoras e validado por 11 especialistas de cinco universidades diferentes: Universidade Federal do Rio Grande, Universidade Federal de Pelotas, Universidade Federal de Ciências da Saúde de Porto Alegre, Universidade Federal de Santa Catarina e Universidade de São Paulo. Eles atuavam nas temáticas de segurança do paciente, tecnologia educacional, infecções relacionadas à assistência à saúde e metodologia CAP.

Na entrega do guia digital, os estudantes foram orientados a lê-lo e a comparar seus conhecimentos prévios com as informações apresentadas, identificando práticas que deveriam ser ajustadas após a leitura. As pesquisadoras informaram que os contatariam novamente em 14 dias, convidando-os para participar da última etapa da pesquisa.

### Terceira etapa da pesquisa

A coleta de dados dessa etapa durou 40 dias e teve a participação de 56 estudantes. No estudo piloto, o tempo médio de resposta do questionário foi de nove minutos. Aplicou-se um instrumento elaborado pelas próprias autoras, constituído por dois blocos: o primeiro continha questões de caracterização sociodemográfica, e o segundo trazia questões sobre o guia digital. Das 28 questões sobre o guia, 21 eram objetivas, com alternativas de “Sim”, “Não” e “Não sei”, referentes às observações dos estudantes sobre o conteúdo no tocante ao conhecimento, atitude e prática; e 7 questões eram de múltipla escolha, referentes aos tópicos abordados em cada capítulo do guia.

Uma das questões tratou especificamente sobre higiene de mãos, buscando compreender quais assuntos abordados haviam sido mais significativos ou acrescentado alguma informação nova para o estudante. As opções de resposta eram: “Os momentos de higiene de mãos”, “As técnicas de higiene das mãos (com solução alcóolica/com água e sabão)”, “Os cuidados com as mãos” e “Não sei dizer”.

As demais seis questões relacionavam-se aos EPIs, buscando identificar quais informações haviam sido mais significativas ou haviam acrescentado um novo conhecimento. Para essas questões, as opções de respostas eram: “Indicação de uso”, “Como calçar e/ou remover”, “Local de descarte” / “Forma de limpeza e desinfecção” e “Não sei dizer”. Ao fim, três questões descritivas e não obrigatórias foram disponibilizadas para que o acadêmico pudesse tecer comentários acerca do guia digital recebido ou da pesquisa realizada.

### Análise dos dados

Os dados desta pesquisa foram tabulados no *Microsoft Excel* ^®^ e transferidos para o *software Statistical Package for the Social Sciences* (SPSS^®^), versão 22. No Excel, analisaram-se os escores do QCPP e QAPP. No SPSS, os dados foram analisados pelo método descritivo de frequências absoluta e relativa, bem como desvio-padrão.

## RESULTADOS

Na primeira etapa da pesquisa, participaram 144 estudantes: 12 (8,3%) eram do 2º semestre; 23 (16%), do 3º semestre; 18 (12,5%), do 4º semestre; 18 (12,5%), do 5º semestre; 14 (9,7%), do 6º semestre; 24 (16,7%), do 7º semestre; 18 (12,5%), do 8º semestre; e 17 (11,8%) do 9º semestre.

No que tange à avaliação do conhecimento dos estudantes, o escore médio foi de 16,67, correspondendo a 83,3% de acertos, considerado um bom conhecimento. Observa-se, nesse resultado, a predominância de acertos nas questões referentes à higiene de mãos e ao uso de luva. Quando analisadas as demais questões, nota-se que apenas 28,5% dos participantes sabiam sobre a necessidade de aderir às medidas de precaução de gotículas, associadas às precauções-padrão, para prestar assistência de enfermagem a pacientes com varicela ou tuberculose ativa. Os demais resultados sobre o conhecimento dos estudantes acerca das precauções-padrão encontram-se na Tabela 1.

**Tabela 1:** Conhecimento dos estudantes de Enfermagem sobre as medidas de precaução-padrão (N = 144), Rio Grande, Rio Grande do Sul, Brasil, 2024

Questão	Verdadeiro n (%)	Falso n (%)	Não sei n (%)	Total N (%)
Você sabe o que são as medidas de precaução-padrão?	127 (88,2)	0 (0)	17 (11,8)	144 (100)
As precauções-padrão só devem ser aplicadas em pacientes com diagnóstico de infecção ou pacientes que se encontrem no período de incubação para uma determinada infecção.	17 (11,8)	119 (82,6)	8 (5,6)	144 (100)
A adesão às medidas de precauções-padrão tem como objetivo principal proteger a equipe de saúde.	110 (76,4)	28 (19,4)	6 (4,2)	144 (100)
Ao entrar em contato com sangue ou quaisquer outros materiais potencialmente contaminados, deve-se lavar as mãos imediatamente.	140 (97,2)	2 (1,4)	2 (1,4)	144 (100)
A higienização das mãos deve ser realizada durante a prestação de cuidados a pacientes diferentes.	135 (93,8)	8 (5,6)	1 (0,7)	144 (100)
Uma vez que o uso de luvas pode evitar a contaminação das mãos, não é necessário higienizá-las depois de retirar as luvas.	4 (2,8)	140 (97,2)	0 (0)	144 (100)
Deve ser evitado o contato de objetos, materiais, equipamentos, roupas e indivíduos com EPI contaminado.	141 (97,9)	2 (1,4)	1 (0,7)	144 (100)
O uso do EPI não deve ser compartilhado.	138 (95,8)	4 (2,8)	2 (1,4)	144 (100)
Ao realizar os procedimentos de cuidado oral ou outros procedimentos que possam envolver contato com mucosas do paciente, o uso de luvas não é obrigatório.	2 (1,4)	142 (98,6)	0 (0)	144 (100)
Nos procedimentos de coleta de sangue ou punção venosa, o uso de luvas é necessário.	143 (99,3)	1 (0,7)	0 (0)	144 (100)
Nos procedimentos em que ocorram possibilidades de contato das mãos com secreção ou excreção de pacientes, o uso das luvas é necessário.	144 (100)	0 (0)	0 (0)	144 (100)
Deve-se trocar as luvas na prestação de cuidados a pacientes diferentes.	144 (100)	0 (0)	0 (0)	144 (100)
Nos procedimentos em que ocorrem possibilidades de respingos de sangue, fluido corporal, secreção ou excreção, deve-se usar máscara de proteção ou protetor facial.	142 (98,6)	1 (0,7)	1 (0,7)	144 (100)
Nos procedimentos em que ocorram possibilidades de respingos de sangue, fluido corporal, secreção ou excreção, deve-se usar óculos de proteção individual ou protetores faciais.	140 (97,2)	2 (1,4)	2 (1,4)	144 (100)
Nos procedimentos em que ocorram possibilidades de respingos de sangue, fluido corporal, secreção ou excreção, deve-se utilizar o avental impermeável de proteção.	140 (97,2)	1 (0,7)	3 (2,1)	144 (100)
Nas situações em que ocorram possibilidades de respingos de sangue, fluido corporal, secreção ou excreção, devem-se utilizar gorros e propés descartáveis.	114 (79,2)	14 (9,7)	16 (11,1)	144 (100)
É proibido dobrar, entortar ou realizar o encape ativo de agulhas. Quando necessário, realizar o encape passivo com apenas uma das mãos. Os recipientes para descarte devem ficar próximos à área de manipulação.	109 (75,7)	19 (13,2)	16 (11,1)	144 (100)
Ao prestar assistência de enfermagem a pacientes com hepatite C ou sífilis, é necessário adotar apenas as medidas de precauções-padrão.	82 (56,9)	34 (23,6)	28 (19,4)	144 (100)
Ao prestar assistência de enfermagem a pacientes com tuberculose ativa ou varicela, é necessário adotar as medidas de precauções-padrão, além das medidas de precaução de gotícula.	92 (63,9)	41 (28,5)	11 (7,6)	144 (100)
Ao prestar assistência de enfermagem a pacientes com infecções intestinais ou infecções de pele, é necessário adotar as medidas de precauções-padrão, além das medidas de precaução de contato.	124 (86,1)	9 (6,3)	11 (7,6)	144 (100)

Quanto à adesão dos estudantes as precauções-padrão, o escore médio foi de 71,5 pontos (89,9%) de acertos, considerada uma boa taxa de adesão. Esse escore, assim como o do conhecimento, é novamente sustentado pelas práticas do uso de luva, cuja pontuação atingiu mais que 90% de acertos na maioria das questões sobre esse tema. Já a utilização de óculos de proteção, de gorro e avental, diante da possibilidade de contato com respingo de sangue, fluido corporal, secreção ou excreção, apresenta, respectivamente, uma taxa de adesão de 48,6%, 38,9% e 53,3%. Os demais resultados sobre a adesão às precauções-padrão encontram-se na Tabela 2.

**Tabela 2:** Adesão dos estudantes de Enfermagem às medidas de precaução-padrão (N = 144), Rio Grande, Rio Grande do Sul, Brasil, 2024

Questão	Sempre n (%)	Frequentemente n (%)	Às vezes n (%)	Raramente n (%)	Nunca n (%)	Total N (%)
Higienizo as mãos no intervalo entre a prestação de cuidados a diferentes pacientes.	115 (79,9)	25 (17,4)	2 (1,4)	1 (0,7)	1 (0,7)	144 (100)
Higienizo as mãos após retirar as luvas.	111 (77,1)	26 (18,1)	6 (4,2)	0 (0)	1 (0,7)	144 (100)
Lavo as mãos imediatamente após contato com material biológico potencialmente contaminado.	138 (95,8)	4 (2,8)	1 (0,7)	0 (0%)	1 (0,7)	144 (100)
Relate **a frequência do uso das luvas** nos procedimentos em que há possibilidade de contato com material biológico potencialmente contaminado, listados abaixo:						
Coleta de sangue ^*^	139 (96,5)	3 (2,1)	0 (0)	0 (0)	1 (0,7)	143 (99,3)
Procedimentos que envolvam a possibilidade de contato com urina/fezes ^*^	138 (95,8)	4 (2,8)	0 (0)	0 (0)	1 (0,7)	143 (99,3)
Procedimentos que envolvam a possibilidade de contato com a pele não íntegra do paciente ^*^	131 (91,0)	9 (6,3)	2 (1,4)	0 (0)	1 (0,7)	143 (99,3)
Procedimentos que envolvam a possibilidade de contato com a mucosa do paciente ^*^	135 (93,8)	4 (2,8)	3 (2,1)	0 (0)	1 (0,7)	143 (99,3)
Procedimentos que envolvam a possibilidade de contato com secreções das vias aéreas do paciente ^*^	135 (93,8)	5 (3,5)	2 (1,4)	0 (0)	1 (0,7)	143 (99,3)
Injeção intramuscular ou subcutânea ^*^	107 (74,3)	23 (16,0)	9 (6,3)	2 (1,4)	2 (1,4)	143 (99,3)
Realização de curativos ^**^	137 (95,1)	4 (2,8)	0 (0)	0 (0)	1 (0,7)	142 (98,6)
Limpeza para a remoção de sangue ^**^	135 (93,8)	5 (3,5)	0 (0)	1 (0,7)	1 (0,7)	142 (98,6)
Punção venosa ^****^	132 (91,7)	7 (4,9)	0 (0)	0 (0)	1 (0,7)	140 (97,2)
Contato com amostras de sangue ^**^	130 (90,3)	10 (6,9)	1 (0,7)	0 (0)	1 (0,7)	142 (98,6)
Utilizo máscara de proteção quando há possibilidade de contato com respingo de sangue, fluido corporal, secreção ou excreção.	112 (77,8)	18 (12,5)	9 (6,3)	2 (1,4)	3 (2,1)	144 (100)
Utilizo óculos de proteção quando há possibilidade de contato com respingo de sangue, fluido corporal, secreção ou excreção.	70 (48,6)	25 (17,4)	25 (17,4)	19 (13,2)	5 (3,5)	144 (100)
Utilizo avental de proteção quando há possibilidade de contato com respingo com sangue, fluido corporal, secreção ou excreção.	77 (53,5)	38 (26,4)	19 (13,2)	9 (6,3)	1 (0,7)	144 (100)
Utilizo gorro e propés descartáveis de proteção quando há possibilidade de contato com respingo com sangue, fluido corporal, secreção ou excreção.	56 (38,9)	31 (21,5)	34 (23,6)	15 (10,4)	8 (5,6)	144 (100)
Não realizo o encape ativo de agulhas usadas ou realizo o encape passivo de agulhas apenas com uma mão.	95 (66,0)	23 (16,0)	6 (4,2)	6 (4,2)	14 (9,7)	144 (100)
Descarto as agulhas, lâminas e outros materiais perfurocortantes em recipientes de descarte específicos.	138 (95,8)	3 (2,1)	1 (0,7)	0 (0)	2 (1,4)	144 (100)
Após acidentes de trabalho com materiais perfurocortantes contaminados, espremo imediatamente o local; em seguida, lavo-o, efetuo a antissepsia e coloco um curativo. ^***^	64 (44,4)	20 (13,9)	4 (2,8)	10 (6,9)	43 (29,9)	141 (97,9)

* Um participante não respondeu à questão;

** dois não responderam à questão;

*** três não responderam à questão;

**** quatro não responderam à questão.

Os escores de 17/20 no conhecimento e 72/80 na adesão foram positivos, mas, em uma análise minuciosa das respostas aos questionários, observaram-se algumas lacunas, tais como: apesar de 88,2% dos estudantes referirem saber o que são as medidas de precauções-padrão, apenas 56,9% concordaram que estas são suficientes para prestar assistência a pacientes com hepatite C ou sífilis. Além disso, 64,3% de estudantes concordaram que, para prestar assistência a pacientes com varicela ou tuberculose ativa, é preciso adotar precaução de gotícula, associada à precaução-padrão; e 66% afirmaram que sempre descartam agulhas utilizadas sem reencapá-las.

Ao fim da primeira etapa, entregou-se o guia digital aos estudantes. Quatorze dias após, iniciou se a terceira etapa da pesquisa, que contou com 56 participantes: um do 2º e um do 3º semestre; dois do 4º e dois do 5º semestre; sete do 6º semestre; 18 do 7º semestre; 15 do 8º semestre; e 10 estudantes do 9º semestre. Entre eles, 92,9% tinham entre 18 e 30 anos.

Após a entrega do guia digital, 96,4% dos estudantes afirmaram que o guia qualificou seu conhecimento, 87,5% indicaram modificação de atitude com base nele, e 92,9% assinalaram que ele permitiu qualificar a prática. Sobre os temas abordados, o mais significativo para os estudantes foi o capítulo de higiene de mãos, com 100% de concordância de que esse conteúdo permitiu aprimorar seus conhecimentos. “Os momentos de higiene de mãos” e “As técnicas de higiene de mãos” foram temas com maior contribuição na qualificação do conhecimento, apresentando, respectivamente, 73,2% e 57,1% de concordância entre os estudantes. Os demais tópicos também alcançaram alta concordância sobre ter havido qualificação do conhecimento. Informações sobre luvas, óculos, gorro, acidente com material biológico e limpeza e desinfecção de superfícies permitiram melhorar o conhecimento de 98,2% dos estudantes. Sobre possíveis mudanças na prática após a leitura do guia, a prática de higiene de mãos e de limpeza e desinfecção de superfícies foram as ações que tiveram mais relatos positivos, com 87,5% em ambas. Em seguida, 85,7% referiram mudanças nas práticas de segregação de resíduos. Em contraste, a menor quantidade de respostas positivas refere-se ao uso de óculos/protetor facial, com 62,5% dos estudantes referindo aumento na frequência de uso.

## DISCUSSÃO

As medidas de precaução-padrão precisam fazer parte do processo formativo de enfermeiros: devem ser inseridas na grade curricular do curso e trabalhadas sistemática e transversalmente^(16,17)^. O foco na qualificação do conhecimento de estudantes de Enfermagem é condição para uma prática segura^(16)^, tanto na forma síncrona quanto por meio de outros recursos e ferramentas educativas^(18)^. É graças ao conhecimento teórico adquirido que estudantes expandem a percepção para tomar decisões clínicas^(19)^. Pesquisas de autorrelato têm sido amplamente aplicadas para avaliar o conhecimento sobre as diretrizes de precauções-padrão e a adesão a elas^(18)^. Ainda que esses trabalhos evidenciem resultados gerais satisfatórios, é essencial explorar o conhecimento dos participantes, visando garantir que um resultado positivo não esconda fragilidades em algumas dimensões^(20)^.

Nosso estudo refletiu essa conduta de exploração do conhecimento ao observar as diferenças entre alto escore de conhecimento e baixa adesão em questões complementares: por exemplo, referir saber quando utilizar as precauções-padrão, mas não identificar ações necessárias em situações específicas, como no atendimento de paciente com varicela ou tuberculose ativa. Esse resultado evidencia falta de conhecimento de mais de 30% dos estudantes a respeito das vias de transmissão de infecção e das indicações de EPIs para cada tipo de exposição.

A varicela e a tuberculose ativa são doenças transmitidas por aerossol, por isso exigem o uso de N95/PFF2 - os únicos equipamentos capazes de proteger as vias respiratórias da inoculação de micropartículas^(21,22)^. Um estudo realizado na Índia com estudantes de Enfermagem e Medicina apresenta semelhante fragilidade no conhecimento, ao evidenciar que apenas 14% dos estudantes de Enfermagem elegeram o respirador “N95” como equipamento adequado contra doenças transmitidas por aerossol e recomendado para ser utilizado em procedimentos geradores de aerossóis^(3)^. Para que um EPI seja adequadamente selecionado, considerando a indicação de uso conforme a assistência a ser prestada, é necessária uma avaliação dos riscos de exposição. Se a avaliação é incorreta, o cumprimento das medidas de proteção torna-se falho, podendo resultar em uma exposição ocupacional^(3)^. O EPI é um recurso fundamental na prevenção de infecções^(20)^; entretanto, apesar da sua essencialidade, este estudo revela uma baixa adesão aos equipamentos, exceto a luva. O percentual de estudantes que referiu sempre utilizar gorro, óculos e avental é de 51,8%, 55,4% e 68,3%, respectivamente. Contudo, 100% dos estudantes afirmaram ter conhecimento da indicação dos óculos de proteção, assim como 98,2% conheciam a necessidade do uso do avental e 85,7% sabiam da indicação de uso de gorro, expondo a realidade de que esse cenário não ocorre por falta de conhecimento.

Um estudo realizado na Arábia Saudita e outro no Brasil apresentam resultados semelhantes no tocante às maiores e menores taxas de adesão aos EPIs. A maior adesão em ambos os estudos foi à luva, seguida da máscara, enquanto a menor adesão foi ao avental e aos óculos/protetor facial^(23,24)^. A luva é o EPI com maior autorrelato de adesão no estudo aqui realizado, variando de 87,5% a 100% para a maioria dos momentos recomendados para uso. Nesse caso, a adesão e o conhecimento apresentam compatibilidade, visto que 100% dos estudantes tinham conhecimento acerca da necessidade de utilizar esse equipamento diante de possibilidades de contato das mãos com secreções ou excreções de pacientes, assim como também sabiam da necessidade de trocar de luvas para atender diferentes pacientes.

Aparentemente, a utilização de luvas está fortemente enraizada nas recomendações de prestação da assistência, enquanto os demais equipamentos sugerem ser indicados apenas para atividades com maior risco de exposição^(25)^. Contudo, a exposição a agentes potencialmente infectantes pode ocorrer na mesma proporção entre as diferentes vias de contato. Logo, diante de respingo de fluidos, não se devem negligenciar os riscos de acometimento dos olhos, nariz ou boca, visto que, independentemente da via de exposição, os acidentes com materiais potencialmente infectantes podem gerar consequências graves aos trabalhadores^(24)^. Os acidentes com material biológico por meio de mucosas é uma realidade, como demonstrou um estudo realizado em Minas Gerais, no qual, dos 111 registros de acidentes analisados, 38% deles correspondiam à exposição de mucosas, que poderiam ter sido evitados por meio do uso do equipamento adequado^(26)^.

Todavia, nos ambientes de práticas, compreende-se que os estudantes são influenciados por diversos profissionais. Dessa forma, é possível que a baixa adesão a alguns equipamentos possa derivar da observação do não emprego desses recursos por parte de outros profissionais. Talvez o acadêmico acredite que, se este, já habilitado, não utiliza determinado equipamento em suas atividades, é porque o seu uso não é necessário^(20)^. Dessa forma, compreende-se que a presença de modelos negativos prejudica significativamente a adesão às precauções-padrão pelos estudantes^(25)^. Esse problema requer intervenções precoces, visando fortalecer as recomendações das medidas de precaução-padrão e incentivar uma adequada conformidade.

Para reduzir essa influência, o educador é fundamental nesse processo, pois é ele quem orienta e direciona os estudantes às melhores práticas. Contudo, para que a atribuição desse educador seja efetiva, é preciso que ele se torne um modelo de conformidade com as precauções-padrão^(25)^. O educador também pode adotar estratégias, como lembrar frequentemente os estudantes, em suas atividades práticas, sobre as medidas que eles devem seguir para evitar infecções^(16)^, ou ainda, periodicamente, incentivar a reflexão sobre a relevância da adesão às precauções-padrão^(25)^. Essas condutas são formas de aumentar a adesão dos estudantes às diretrizes de precauções-padrão^(27)^.

O reforço da importância dessas medidas pode ocorrer tanto por monitoramento das ações associado a feedbacks quanto por métodos teóricos. Quando uma liderança organizacional mais visível aos estudantes é associada às estratégias para promover altos níveis de adesão, a prática do conhecimento teórico é favorecida^(27)^. Dentre as estratégias que vêm apresentando bons resultados para esse objetivo, está o emprego da tecnologia no processo de ensino e aprendizado, devido ao potencial dela de melhorar ou até mesmo acelerar o desenvolvimento estudantil. Muito desse benefício é influenciado pelo atual perfil dos estudantes. Dessa forma, considerar qual geração de estudantes predomina no ensino superior é fundamental para pensar em modelos educacionais^(28)^. Atualmente as gerações de estudantes mais prevalentes no ensino superior são a Geração Y e a Geração Z^(29)^, conforme os resultados do estudo que indica 94,6% dos participantes tendo de 18 a 40 anos.

Apesar de suas particularidades, ambas as gerações fazem uso de tecnologias. Os estudantes da Geração Z, por serem da primeira geração a ter nascido em um contexto de tecnologia amplamente utilizada^(28,29)^, apresentam um desenvolvimento muito mais significativo nesse cenário. No ensino superior, esses estudantes preferem aprender por meio de materiais eletrônicos e audiovisuais. Recursos como o aplicativo WhatsApp têm ampla adesão entre esse público, porque facilitam o compartilhamento de informações acadêmicas com os colegas. O atrativo para eles é a disponibilidade dos recursos digitais de forma ininterrupta^(28)^. Logo, foi por perceber as preferências dessa geração e a importância da tecnologia para a educação, que o estudo desenvolvido disponibilizou um guia digital. Considerou-se que ele seria uma proposta adequada para qualificar o conhecimento e incentivar a maior adesão às precauções-padrão.

Utilizar tecnologias no processo educativo tem demonstrado avanços, mesmo que pequenos, nos resultados educacionais relacionados à intervenção^(28)^. No nosso estudo, também houve essa identificação, observando-se resultados positivos após a entrega do guia digital. Suas informações objetivas e interativas contribuíram para com o ensino teórico-prático dos estudantes, qualificando o conhecimento, incentivando atitudes positivas e guiando a prática. A sustentação das competências necessárias para prevenir e controlar infecção exige esforços, tempo^(17)^ e percepção do risco a que se está exposto, sendo este um fator importante para a atitude de aderir às medidas de precaução^(30)^. Possivelmente por esse motivo, a concordância de que o guia digital incentivou atitudes positivas foi a menor entre as três avaliações. Ainda assim, alcançou 87,2%, abaixo dos percentuais atribuídos à qualificação do conhecimento e da prática.

O tempo entre adquirir conhecimentos com o guia e pôr em prática esses conhecimentos pode ter influenciado a compreensão dos riscos e a percepção de estar em risco. Dessa forma, é preciso desenvolver estudos para confrontar o comportamento autorreferido com a observação na prática. A estratégia deve seguir sendo implementada e multiplicada entre os estudantes do curso.

### Limitações do estudo

Este estudo foi desenvolvido no estado do Rio Grande do Sul, durante um calendário acadêmico atípico, marcado por eventos climáticos extremos. O resultado desse contexto foram diversas interrupções nos dias letivos e a diminuição do tempo para desenvolver a pesquisa, comprometendo mais significativamente a terceira etapa. Outra limitação encontrada foi a baixa adesão dos estudantes, especialmente de anos iniciais, a essa terceira etapa, impedindo comparações entre os semestres. A razão para esse fato pode estar em fatores como frágil conhecimento sobre o desenvolvimento de pesquisas, pouca exposição do conteúdo em disciplinas teóricas e pouco contato com a prática em cenários de serviço de saúde. As limitações nos mostraram que mudanças pedagógicas precisam ser realizadas no curso e novos estudos precisam ser levados em consideração para possibilitar comparações.

### Contribuições para a Área

Este estudo disponibilizou um guia digital para a temática das precauções-padrão, visando qualificar o conhecimento e incentivar a maior adesão a essas medidas. Além disso, foi possível apontar diversas formas para trabalhar assertivamente as ações necessárias para minimizar a exposição ocupacional vivenciada atualmente pelos estudantes.

## CONCLUSÕES

Este estudo permitiu identificar lacunas no conhecimento, formas de incentivar atitudes positivas, práticas que estavam em conformidade com as recomendações e práticas que necessitavam de intervenções. O guia digital trouxe contribuições positivas aos estudantes. Portanto, sugere-se o uso contínuo de diferentes tecnologias educacionais durante o processo formativo, sanando possíveis dúvidas e promovendo a adesão dos estudantes de Enfermagem às precauções-padrão. Ainda, recomenda-se o estímulo ao pensamento crítico, evidenciando aos estudantes os riscos e consequências quando as diretrizes não são seguidas. Dessa forma, pode-se favorecer a conscientização sobre a importância de cada diretriz de precaução-padrão.

## Data Availability

Os dados de pesquisa estão disponíveis no corpo do artigo.
